# Hip Pain Due to Leiomyoma After Total Hip Arthroplasty: A Case Report

**DOI:** 10.7759/cureus.95833

**Published:** 2025-10-31

**Authors:** Stavros Lykos, Zoi Frida, Konstantinos Tsivelekas, Dimitrios Pallis, George Macheras, Stamatios A Papadakis

**Affiliations:** 1 Second Department of Orthopedics, KAT General Hospital of Attica, Athens, GRC; 2 Seventh Department of Orthopedic Surgery, Henry Dunant Hospital Center, Athens, GRC

**Keywords:** chronic hip pain, extrauterine fibroid, leiomyoma, obturator neuropathy, total hip arthroplasty (tha)

## Abstract

Total hip arthroplasty (THA) is a widely recognized treatment for various hip disorders, significantly improving pain and function. Despite advances in surgical techniques, a proportion of patients experience postoperative pain, leading to a necessity for revision. This report outlines a unique case of chronic hip pain post-THA associated with the unexpected identification of a large leiomyoma, an occurrence not yet reported in the literature. A 75-year-old female with a history of right THA presented with persistent left hip pain two years after the operation. After left hip replacement, she initially reported significant improvement, only to experience severe pain six months later. Diagnostic imaging with MRI revealed a leiomyoma close to the left hip, suspected of causing obturator neuropathy. Following interdisciplinary consultation, surgical removal of the fibroid was performed, resulting in complete resolution of symptoms. The patient underwent an evaluation two years following the surgical removal of the fibroid. At this juncture, her Harris Hip Score was recorded at 87, and she was completely free from pain.

## Introduction

Total hip arthroplasty (THA) is recognized as a highly effective treatment for alleviating pain and improving function across a range of hip pathologies. Commonly used to address conditions such as osteoarthritis, osteonecrosis, and post-traumatic hip arthritis, THA plays a crucial role in orthopedic care. In the United States alone, the importance of joint replacement surgeries is evident from the significant annual count of over 450,000 hip replacements, a number that continues to rise in correlation with an aging population [1]. Notably, these procedures have a long-lasting impact, with hip replacements often enduring for more than 20 years, offering most patients a permanent solution to hip arthritis [1,2].

Despite advancements in surgical techniques and implant quality over the past two decades, a notable proportion of patients, approximately 27%, report pain during the first six months post-operation [2]. This issue can escalate, leading up to 4% of patients to suffer from severe chronic pain, eventually requiring revision surgery [2,3]. These findings underscore the necessity for healthcare professionals to perform thorough assessments of THA patients to identify and address any underlying complications. In cases of revision THA, Bozic et al. (2009) identified the most commonly reported causes for such procedures, including instability or dislocation, mechanical loosening, infection, and periprosthetic fractures [3]. Beyond these common issues, there are other, more rare complications, such as iliopectineal bursitis and soft tissue, bony, neurological, vascular, or psychological causes [4,5].

Often, a multidisciplinary approach is required for diagnosis and management to effectively alleviate chronic pain in patients with a stable prosthesis. Making an accurate diagnosis is essential for successful treatment, which might be non-operative or operative [2]. Here, we present a particularly unique case of an encountered leiomyoma that resulted in chronic hip pain following THA. To our knowledge, a review of the available bibliographic literature revealed no documented instances of such an occurrence.

Uterine fibroids, also known as leiomyomata or myomas, represent the most prevalent non-cancerous tumors in women. Baird et al. (2003) revealed that by the age of 50 years, the projected occurrence of fibroids was 70% among white women [6]. These fibroids develop from the smooth muscle cells of the uterus (myometrium), and their growth is largely influenced by the amount of estrogen in the bloodstream [7]. Uterine fibroids are commonly found in three primary areas, namely, subserosal (on the outer surface of the uterus), intramural (within the myometrium), and submucosal (inside the uterine cavity) [8]. Extrauterine leiomyomas, which are less common, pose a more significant challenge in diagnosis. Originating from smooth muscle cells, these histologically benign tumors predominantly develop within the genitourinary tract, including areas such as the vulva, ovaries, urethra, and urinary bladder, yet they can emerge in almost any anatomical location. Moreover, they may exhibit atypical growth behaviors, such as benign metastasizing leiomyoma, disseminated peritoneal leiomyomatosis, intravenous leiomyomatosis, parasitic leiomyoma, and growth in the retroperitoneal space [8].

## Case presentation

A 75-year-old female patient presented at the outpatient clinic of our hospital, reporting persistent anterior left hip pain that had persisted for over a year. The patient described the pain as constant, exacerbating notably with physical exertion, such as ascending stairs, significantly impairing her ability to perform daily activities. Her medical history was largely unremarkable, with no noteworthy conditions or regular medication usage, except for treatment of arterial hypertension. Two years prior, the patient had undergone a successful right THA, which effectively alleviated the pain in that hip.

During the clinical examination, the patient exhibited a reduced range of motion and demonstrated weakness in the hip abductors. Notably, there was a limitation in external rotation, with the patient unable to achieve rotation beyond 10 degrees. The patient’s modified Harris Hip Score was 72. The patient experienced pain upon active hip extension, accompanied by crepitus during movement. Additionally, she was unable to rise from a seated position in a chair without using her hands for assistance. Radiological and paraclinical assessments showed a well-functioning total hip replacement on the right side without any signs of loosening, along with severe osteoarthritis observed in the left hip. Based on these findings, a decision was made to proceed with surgical intervention for the left hip, and the patient provided written informed consent.

The left hip replacement surgery was conducted two months following her initial presentation. The postoperative period was uneventful, and the patient was discharged three days after the surgery. The patient was systematically evaluated at intervals of one, three, and six months following the surgical procedure. At the three-month mark postoperatively, the patient reported no pain, had returned to all prior physical activities, including the ability to ascend stairs, and could rise from a seated position without the assistance of her hands with a Harris Hip Score of 82.

**Figure 1:**
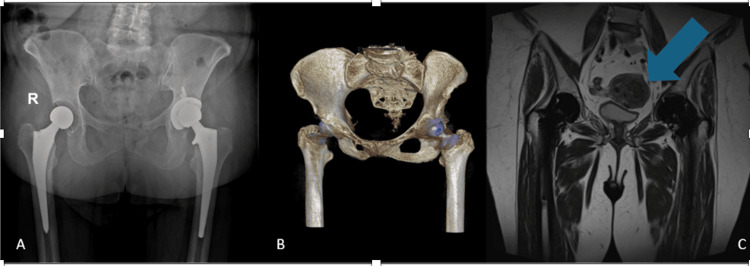
(A) Postoperative anteroposterior pelvic radiograph. (B) Pelvic three-dimensional CT scan. (C) Pelvic MRI. The arrow reveals a large leiomyoma in proximity to the left hip.

However, at the six-month evaluation, the patient presented with complaints of severe pain that had emerged over the preceding month and was escalating in severity. This pain was impeding her range of motion, and she had resumed using a walking frame due to difficulties with ambulation. Radiological evaluations (Figure 1) at this time showed no signs of loosening in the left hip, and inflammatory markers, including C-reactive protein and erythrocyte sedimentation rate, were within normal limits (Table 1). An MRI scan was subsequently performed, revealing a large leiomyoma in proximity to the left hip (Figure 1). Suspecting that the leiomyoma was leading to compressive obturator neuropathy, the patient was then referred to her gynecologist for further evaluation.

**Table 1:** Laboratory findings at the time of symptom presentation.

Parameter	Patient value	Units	Reference range
White blood cell count	7.4	×10⁹/L	4.0–10.0
Hemoglobin	12.8	g/dL	12.0–16.0
C-reactive protein	<0.32	mg/L	<0.32
Erythrocyte sedimentation rate	12	mm/hour	<20
Platelet count	250	×10⁹/L	150–400

After the appropriate gynecological examination, a recommendation was made for the surgical removal of the fibroid, to which the patient consented. She was discharged one day post-surgery, reporting no pain and a full range of motion in the left hip as early as five days after the operation. Two years post-surgery, the patient remains pain-free with a Harris Hip Score of 87. This indicates that the chronic hip pain post-THA was likely due to the large leiomyoma causing obturator neuropathy.

## Discussion

Leiomyomas involving the hip region are exceptionally rare, particularly following THA. Only a few cases of leiomyoma or leiomyosarcoma developing adjacent to hip prostheses have been reported in the literature, most of which presented with non-specific symptoms such as pain or restricted motion [1-3]. In most reported cases, the differential diagnosis initially included periprosthetic infection or mechanical loosening, and the correct diagnosis was established only after advanced imaging or biopsy.

In the present case, the leiomyoma developed in close proximity to the left hip prosthesis, causing pain and impaired ambulation without evidence of infection or prosthetic loosening. Similar to previously described cases, MRI played a crucial role in delineating the lesion and excluding prosthetic complications. However, unlike the majority of reported cases, our patient’s inflammatory markers remained within normal limits, highlighting the importance of considering soft tissue tumors in the differential diagnosis of unexplained post-THA pain.

Although the pathogenesis of tumor formation adjacent to orthopedic implants remains uncertain, previous research has suggested that chronic irritation or particulate debris from metallic prostheses may induce local inflammatory or mutagenic effects, potentially contributing to neoplastic transformation [9]. In addition, rare cases of sarcoma developing near implants have been described, supporting the hypothesis that long-term exposure to corrosion products or mechanical friction could play a role in tumorigenesis [10].

This case underscores the need for clinicians to maintain a broad diagnostic perspective when evaluating post-arthroplasty pain. Awareness of rare entities such as leiomyoma can facilitate earlier diagnosis and appropriate referral, ultimately improving patient outcomes.

## Conclusions

This case describes an exceptionally rare cause of chronic hip pain following THA, resulting from a large leiomyoma compressing the obturator nerve. It highlights the need to consider uncommon extra-articular and non-orthopedic causes when persistent pain occurs after THA and standard imaging reveals no abnormalities. Awareness of such rare etiologies can help avoid unnecessary revision procedures. The patient’s full recovery after leiomyoma excision demonstrates the value of multidisciplinary collaboration among orthopedic, radiologic, and gynecologic teams. This case broadens clinical understanding of postoperative hip pain and reinforces the importance of a comprehensive, patient-centered diagnostic approach.
